# Areas of uncertainty on the diagnosis, treatment, and follow-up of hypophosphatemia in adults: an Italian Delphi consensus

**DOI:** 10.1007/s40618-024-02458-4

**Published:** 2024-10-08

**Authors:** Iacopo Chiodini, Daniela d’Angela, Alberto Falchetti, Luigi Gennari, Nazzarena Malavolta, Laura Masi, Antonio Migliore, Massimiliano Orso, Barbara Polistena, Domenico Rendina, Alfredo Scillitani, Federico Spandonaro, Giuseppe Vezzoli, Fabio Vescini, Delphi Panel Members, Maria Rosaria Ambrosio, Elisa Cairoli, Valentina Camozzi, Salvatore Cannavò, Cristina Eller-Vainicher, Sandro Gianninir, Laura Gianotti, Andrea Giusti, Daniela Merlotti, Silvia Migliaccio, Salvatore Minisola, Vincenzo Montinaro, Andrea Palermo, Daniela Pasquali, Giovanni Passeri, Massimo Procopio, Antonio Stefano Salcuni

**Affiliations:** 1 Department of Medical Biotechnology and Translational Medicine, University of Milan, Milan, 20100 Italy; 2 Unit of Endocrinology, ASST Grande Ospedale Metropolitano Niguarda, Milan, Italy; 3 C.R.E.A. Sanità (Centre for Applied Economic Research in Healthcare), Rome, Italy; 4 Department of Medicine, Surgery and Neurosciences, University of Siena, Siena, Italy; 5 Madre Fortunata Toniolo Private Hospital, Bologna, Italy; 6 Metabolic Bone Diseases Unit, University Hospital of Florence (AOUC), Florence, Italy; 7 Department of Clinical Medicine and Surgery, Federico II University, Naples, Italy; 8 U.O. di Endocrinologia, Ospedale “Casa Sollievo della Sofferenza”, IRCCS, San Giovanni Rotondo, FG Italy; 9 University of Rome Tor Vergata, Rome, Italy; 10 Nephrology and Dialysis Unit, IRCCS San Raffaele Scientific Institute, Vita Salute San Raffaele University, Milan, Italy; 11 Endocrinology Unit, University-Hospital of Udine, Udine, Italy

## Abstract

**Purpose:**

The study aimed to present the results of a Delphi consensus involving Italian experts focusing on the management of hypophosphatemia in adults.

**Methods:**

A multidisciplinary advisory board of nine physicians, experts in hypophosphatemia management, was established. Next, a literature search was performed to identify international guidelines, consensus, and clinical pathways, which were later presented to the advisory board. Collaboratively, the advisory board and authoring team selected key statements for the consensus process and focused on areas of uncertainty related to the management of hypophosphatemia. The advisory board also indicated the experts to be invited to participate in the consensus process. The Delphi method was employed to reach a consensus.

**Results:**

The literature search yielded one guideline, five consensus documents, and one clinical pathway. While our search strategy aimed to identify documents on the management of all types of hypophosphatemia, most of the guidelines and consensus documents retrieved focused on X-linked hypophosphatemia. The consensus process focused on 11 key issues, achieving strong convergence (over 70% consensus) in the first Delphi round for 8 out of the 11 statements. Three statements proceeded to the second round, with strong agreement reached for two. Notably, consensus was not reached for the statement concerning the measurement of fibroblast growth factor 23 for diagnostic purposes.

**Conclusion:**

The study revealed that the community of clinical experts is well-informed and in agreement regarding hypophosphatemia management. It emphasized the importance of developing clear national guidance documents to support clinicians and multidisciplinary teams in patient management. These documents are crucial not only for healthcare professionals but also for those responsible for defining pathways and services, facilitating a more accurate management of hypophosphatemic patients.

**Supplementary Information:**

The online version contains supplementary material available at 10.1007/s40618-024-02458-4.

## Introduction

Maintaining extracellular and intracellular phosphate levels within a certain range is vital for the health of the entire organism and has a crucial biological value for bone health. Chronic hypophosphatemia can be caused by several conditions, such as fibroblast growth factor 23 (FGF23)-dependent hypophosphatemia, which can be genetic (caused by mutations of the phosphate regulating gene, PHEX) or acquired, and it is characterized by various symptoms, including reduced absorption of phosphate (due to vitamin D deficiency or resistance) and increased urinary excretion (due to primary or secondary hyperparathyroidism or primary reabsorption deficiency). Among the forms of FGF23-dependent hypophosphatemia with a genetic etiology, the most common is certainly X-linked hypophosphatemia (XLH), which can be considered the prototype of a hereditary disorder caused by loss of phosphate at the renal level. XLH is characterized by skeletal abnormalities of varying severity, growth retardation, rickets and/or osteomalacia, bone pain, enthesopathy, osteoarthritis, spontaneous tooth abscesses, hearing problems, and muscle dysfunction. Hypophosphatemic conditions which are FGF23-independent also exist (e.g., hereditary hypophosphatemic rickets with hypercalciuria, nephrolithiasis) [1].

The lifelong and complex nature of this condition requires an interdisciplinary approach aimed at managing the wide range of symptoms and at maximizing the patients’ quality of life [2].

However, there are other less common forms of hereditary FGF23-dependent hypophosphatemia, such as autosomal dominant hypophosphatemic rickets (ADHR) and recessive (autosomal-recessive hypophosphatemic rickets, ARHR), polyostotic fibrous dysplasia, hypophosphatemic rickets with hyperparathyroidism and non-lethal Raine syndrome [1].

Among the acquired forms of FGF23-dependent hypophosphatemia, there is tumor-induced osteomalacia (TIO), a rare paraneoplastic condition characterized by bone demineralization and a loss of phosphate through the kidney. Typically caused by a phosphaturic mesenchymal tumor of the mixed connective tissue, it can be caused also by other type of tumors (e.g., fibromas, chondrosarcomas, neuroblastomas, osteosarcomas, and soft tissue tumors), most commonly benign, but cases of malignant disease have been found [3, 4]. In adult patients, this results in osteomalacia associated with bone pain, pathological fractures, muscle weakness, and vertebral deformity [5].

In recent years, international guidelines and consensus have been mainly released concerning the management of patients with XLH [6–8], while only consensus documents are available for TIO [9]. This paper describes the results of a Delphi consensus that involved Italian experts on the management of hypophosphatemia in adults. The Delphi process aimed to reach a consensus on the case definition, diagnosis, treatment, and follow-up of adult patients with hypophosphatemia. After examining the international literature, possible areas of uncertainty in the management of hypophosphatemic adult patients were identified. Subsequently, a set of statements related to the identified areas of uncertainty were submitted to the clinical experts in order to assess their level of agreement on the matter.

## Methods

A multidisciplinary advisory board consisting of nine physicians with expertise in the management of hypophosphatemic patients was established. Next, the authoring team conducted a literature search aimed at identifying international guidelines, consensus, and clinical pathways which were later presented to the advisory board. The advisory board, supported by the authoring team, selected the statements for the consensus process.

The same advisory board also indicated the experts to be invited to participate in the consensus process on the statements. The Delphi method was used to reach consensus.

The work was possible thanks to an unconditional contribution from Kyowa Kirin.

## Literature search

A systematic literature search was conducted in PubMed, Embase, Web of Science, Google Scholar, and Google up to July 2022 to identify relevant guidelines, consensus or integrated care pathways on the management of hypophosphatemia in adults. The full search strategy is reported in Online Resource 1. The study selection process was performed independently by two reviewers. The selection of studies was conducted in two phases. Records were initially assessed by screening titles and abstracts, based on predefined inclusion criteria: we included guidelines, consensus statements, integrated care pathways, written in English or Italian, reporting on the diagnosis, treatment and follow-up of adult patients with hypophosphatemia. Later, full-text articles of potential eligible studies were assessed. Disagreements between reviewers were resolved through discussion. The literature selection process is depicted in Online Resource 2 (PRISMA 2020 Flow Diagram).

### Areas of uncertainty

The authoring team, with the advisory board’s support, decided to focus on certain aspects related to the identification of the hypophosphatemic patient, diagnosis, therapeutic options, and patient’s follow-up. From the analysis of the guidelines and subsequent discussion, it was agreed to focus the consensus-seeking process on the following subjects: (1) definition of serum phosphate level thresholds; (2) threshold definition of the ratio of tubular maximum reabsorption of phosphate (TmP) to glomerular filtration rate (GFR) (TmPO4/GFR); (3) diagnostic usefulness of FGF23; (4) definition of FGF23 reference values; (5) definition of vitamin D deficiency in patient with hypophosphatemia; (6) definition of normal urinary calcium excretion values; (7) assessment of phosphorus intake in vegetarian and vegan diets; (8) evaluation of vitamin D levels and burosumab treatment; (9) assessment of physical performance as part of the assessment of the efficacy of therapies; (10) need for radiographic imaging for evaluation of elusive clinical signs; 11) use of Patient-Reported Outcome Measures (PROMs) for functional assessments. After defining a premise and a statement for these subjects, consensus was sought through the Delphi process described below.

### Delphi study design

A two-round Delphi method was used to reach consensus among a panel of clinical experts [10]. This method has been previously used by the authors in similar studies [11]. The Delphi method is a structured technique aimed at leading a group of experts to reach consensus on a complex or uncertain topic through a series of questionnaires interspersed with controlled feedback. The process guarantees the anonymity of individual responses, avoiding possible source of bias due to dominance and group conformity, also known as groupthink [10]. In addition, the method allows respondents to modify their initial judgements after receiving controlled feedback. Finally, respondents can provide comments on statements that do not achieve strong convergence, offering useful insights for the analysis of disagreements [12].

In total, 11 statements (Table 1) were formulated for the purpose of the Delphi process. The panel received an e-mail invitation to participate in the study and completed the survey rounds online. Data were analysed with descriptive statistics. The panel was selected by convenience sampling, using the suggestions from the advisory board members, and consisted of 25 professionals operating in 13 Italian regions.

**Table 1:** Statements proposed to the panel of clinical experts at the first round of the Delphi process

S1	BACKGROUND: Hypophosphatemia in adults is defined as a serum phosphate level less than 2.5 milligrams per deciliter (mg/dL). STATEMENT: Serum phosphate levels below 2.7 mg/dL represent a warning in the concomitant presence of symptoms/signs potentially related to hypophosphatemia.
S2	*BACKGROUND: The ratio between maximal tubular phosphate reabsorption and glomerular filtration rate (TmPO4/GFR) is a crucial parameter for estimating tubular phosphate reabsorption in an individual. In adults*,* the TmPO4/GFR score has reference values of 3.3 ± 0.3 mg/dL (lower limit of normality: 2.7 mg/dL).* **STATEMENT**: TmPO4/GFR scores lower than 2.7 mg/dL in the presence of serum phosphate levels lower than 2.7 mg/dL suggest the start of the diagnostic pathway for a hypophosphatemic patient.
S3	*BACKGROUND: Fibroblastic growth factor 23 (FGF23) mainly regulates the concentration of phosphates in plasma by reducing their tubular reabsorption and increasing their urinary excretion.* **STATEMENT**: FGF23 measurement is considered necessary for the diagnosis and initiation of treatment of hypophosphatemia.
S4	*BACKGROUND: The reference values of FGF23 also differ in relation to the diagnostic kit used and the variable measured (intact FGF23 or C-terminal portion).* **STATEMENT**: FGF23 values should never be considered as an isolated parameter but should always be assessed in relation to phosphate levels.
S5	*BACKGROUND: Vitamin D deficiency*,* if left untreated*,* could lead to the onset of secondary hyperparathyroidism. Correction of vitamin D deficiency is recommended.* **STATEMENT**: A vitamin D level < 30 ng/mL is an indication for its correction in patients with hypophosphatemia.
S6	*BACKGROUND: Therapies based on phosphate and/or vitamin D or its analogues must be defined based on the urinary calcium excretion values which constitute a limit for the administration of calcitriol.* **STATEMENT**: Normal urinary calcium excretion values are less than 250 mg/24 h for women and 300 mg/24 h in men, or 4 mg/kg of body weight in 24 h.
S7	*BACKGROUND: Phosphate is mainly present in foods of animal origin (e.g. fish*,* milk*,* cheese).* **STATEMENT**: As part of a vegetarian/vegan diet, it is necessary to increase the intake of vegetables that provide a sufficient level of phosphate, such as bran, wheat germ, soy.
S8	*BACKGROUND: Reduced vitamin D values were observed in a portion (28%) of patients treated with burosumab.* **STATEMENT**: Adequate vitamin D levels must be maintained throughout the treatment with burosumab.
S9	*BACKGROUND: The evaluation of physical performance is important to detect the effectiveness of therapies which may not necessarily be associated with variations in chemical-clinical parameters but with improvements in the functional sphere.* **STATEMENT**: The use of the hand grip (or other tests) is considered useful for the assessment of muscular strength.
S10	*BACKGROUND: Enthesopathies*,* early osteoarthritis*,* pseudo-fractures and fractures are typical signs of hypophosphatemic subjects and often go unnoticed during clinical and biochemical evaluations.* **STATEMENT**: Radiographic imaging is useful during clinical/therapeutic follow-up for the diagnosis of bone, joint and tendon alterations that the hypophosphatemic subject may develop.
S11	*BACKGROUND: Patient-Reported Outcome Measures are often used in clinical trials for functional assessments and to measure therapeutic outcomes.* **STATEMENT**: The “Western Ontario and McMaster Universities Osteoarthritis Index (WOMAC^®^)” scale and the “Brief Pain Inventory (BPI)” questionnaire can be useful for evaluating health status and treatment outcomes in terms of improvement in stiffness, physical function and pain reduction.

### Questionnaire and survey

The Delphi process was launched on February 1, 2023, and completed on April 23, 2023. Panellists used a dedicated online platform to participate and a timeline of 15 calendar days was set to provide answers for each round. A further 15 days were granted after a reminder e-mail. Two reminders were sent to gather as many responses as possible. The agreement was defined using a 9-point scale where scores from 1 to 3 were used to indicate little or no agreement, scores from 4 to 6 were used to indicate moderate agreement, scores from 7 to 9 were used to indicate full agreement with the proposed statement. The cut-off for consensus was set at a minimum of 70% of the number of respondents, meaning that strong disagreement or agreement was considered reached if at least 70% of participants had assigned scores in the range 1–3 or 7–9 to that statement, respectively [10]. Statements with average score within the intermediate range, meaning 4–6 (corresponding to “moderate agreement”), were not taken as indicative of a strong convergence in terms of agreement/disagreement. As per protocol, the statements for which the level of agreement/disagreement didn’t reach the threshold were submitted for a second round in which only the respondents to the first round were invited to participate. Results of the first round were shared with the respondents.

## Results

### Literature search

The literature search initially identified 555 potentially eligible records. After removal of duplicates, 416 records were examined by title and abstract and, of these, 17 were examined in full text to evaluate their inclusion. The analysis of the full text studies led to the inclusion of a total of 7 studies: 1 guideline [6], 5 consensus [7, 8, 13–15] and 1 clinical pathway of an Italian local health unit [16].

The main characteristics of the included studies are described in Table 2, while the studies evaluated in full-text and excluded are described in Online Resource 3. Remarkably, only one [6] among the included studies, was structured as a guidelines document and had the focus on XLH.

**Table 2:** Main characteristics of included studies

Study	Design	Country	Target population	Clinical area
Haffner 2019 [6]	Guideline	Europe	Children and adults with XLH	Diagnosis, treatment and follow-up
Carpenter 2011 [13]	Consensus	USA	Children and adults with XLH	Treatment
Dahir 2022 [14]	Consensus	USA	Adolescents and young adults (transitional age) with XLH	Management
García Martín 2020 [15]	Consensus	Spain	Patients with hypophosphatemia and hyperphosphatemia	Management
Laurent 2021 [7]	Consensus	Belgium	Children and adults with XLH	Diagnosis, treatment and follow-up
Trombetti 2022 [8]	Consensus	International	Children and adults with XLH	Diagnosis, treatment and follow-up
Baroncelli 2020 [16]	Clinical pathway	Italy	Patients with hypophosphatemic rickets	Diagnosis, treatment and follow-up

### Identification of the hypophosphatemic patient

The following clinical signs and symptoms are commonly recognized as associated with hypophosphatemia [6–8, 14]: history of rickets, growth retardation or deformity of the lower limbs, cranial deformities or other physical deformities, clinical and/or radiological signs of osteomalacia (including pseudo-fractures, early arthrosis and enthesopathies), serum phosphate levels below the age-related reference range, renal phosphate depletion, dental abscesses, periodontal disease, fatigue/weakness/asthenia and/or muscle pain, osteo-articular pain, crooked gait and/or other gait disorders, joint stiffness.

To define the diagnosis, after the medical history and clinical examination, it appears necessary to exclude causes related to blood dilution (e.g. due to massive fluid resuscitation, dialysis, plasmapheresis), spurious hypophosphatemia (e.g. interference of drugs such as amphotericin B, interference by bilirubin or specific paraproteins), effects of drugs (e.g. phosphate binders, niacin) or alcohol abuse [7]. From a diagnostic point of view, it is also essential to discriminate between renal and non-renal causes of hypophosphatemia, measuring the ratio between the maximum tubular reabsorption of phosphate and the glomerular filtration rate (TmPO4/GFR) which must be calculated from fasting plasma samples and fasting spot urine from the second morning void (obtained 2 h after the first voided urine) for measurement of phosphate and creatinine. Recently, Arcidiacono and colleagues demonstrated that TmP/GFR must be effectively calculated also using 24 h urine collection in adult patients with FGF23-dependent renal phosphate leak [17]. This parameter can be obtained using the Walton and Bijvoet nomogram or using the Kenny and Glen algorithm [8]. If renal phosphate wasting is documented, a distinction must be made between hereditary and acquired FGF23-dependent or FGF23-independent conditions. Regarding family history, international guidelines [6] recommend that any first-degree family member of a patient with XLH should be investigated for XLH, even though sons of the males affected by XLH are not at risk. If the PHEX gene analysis produces a negative result for XLH, it is recommended to also evaluate other causes of hereditary or acquired hypophosphatemia. Furthermore, genetic counselling is recommended for patients with XLH, particularly in the transition from paediatric to adult age and for families planning a pregnancy. If genetic analysis is not available, elevated or inappropriately normal plasma levels of intact FGF23 and/or a positive family history of XLH support the diagnosis.

### Diagnostic workout

The following laboratory tests are indicated for the diagnostic evaluation of the XLH patient [6–8, 14]: serum phosphate, serum calcium, bone-specific alkaline phosphatase (ALP), parathyroid hormone (PTH), 25(OH) vitamin D, 1,25(OH)2 vitamin D, FGF23, serum creatinine, urinary calcium: creatinine ratio, urinary phosphate and creatinine levels to be used for the calculation of TmPO4/GFR.

The presence of hypophosphatemia and loss of phosphate through the urine, in the absence of Fanconi syndrome and hereditary hypophosphatemic rickets, suggests the need to look for a neoplasm in the context of oncogenic osteomalacia. Since these mesenchymal tumours usually express somatostatin receptors, their presence can be ascertained by Indium-111-labeled octreotide scintigraphy (Octreoscan™), although 68Gallium DOTATOC positron emission tomography/computed tomography and technetium-99 m HYNIC-TOC single-photon emission computed tomography showed the highest sensitivity [18]. All diagnostic images must be total body (i.e., from head to toe). The differential diagnosis includes other forms of hypophosphatemic osteomalacia (e.g. XLH, autosomal dominant or recessive) and primary or acquired Fanconi renal syndrome. Primary Fanconi syndrome is usually sustained by inherited diseases (cystinosis, Wilson disease, tyrosinemia, galactosemia) and, being typical of the childhood, its main clinical manifestations are related to rachitis and growth defects. On the contrary, the acquired form of Fanconi regards the adults and its main skeletal manifestation is osteomalacia. Both the forms may be associated with asthenia, polyuria, polydipsia and hypovolemia (following ions losses), constipation and muscle weakness (due to hypokalaemia) and sings of hyperchloremic metabolic acidosis in case of rapid onset (headache, lack of energy, nausea, and vomiting).

A series of additional tests can be considered for differential diagnosis [6, 7], including urinary pH, plasma bicarbonate, urinary amino acids, urinary glucose, uric acid in urine, and low molecular weight proteinuria.

Radiological examinations and other tests may be considered as well. It is advisable to perform X-rays of the lower limbs and wrist (including the assessment of bone age) using low radiation dose investigation techniques. DXA is generally not recommended in patients with XLH [7, 14]. International guidelines [6] also recommend carrying out renal ultrasonography, to evaluate the possible presence of stones, and a neurological examination, to evaluate the consequences of craniosynostosis and spinal stenosis.

### Treatment

#### Conventional pharmacological treatment

In symptomatic adults with XLH, international guidelines and consensus [6, 7, 13] recommend treatment with active vitamin D (calcitriol or alfacalcidol) together with oral phosphorus (phosphate salts) to reduce osteomalacia and its consequences and to improve oral health. The recommended dosage is 750–1,600 mg per day (based on elemental phosphorus) for phosphate and 0.50–0.75 and 0.75–1.5 µg per day for calcitriol and alfacalcidol, respectively [6].

However, routine treatment of asymptomatic hypophosphatemic adults, including XLH adult patients, is not recommended [6]. Phosphate supplements should not be prescribed without vitamin D analogues, as phosphate alone promotes secondary hyperparathyroidism and thus renal phosphate wasting [7].

It is recommended to treat pregnant and breastfeeding women with active vitamin D in combination with phosphate supplements [6, 8].

Doses of active vitamin D should be reduced in patients who develop hypercalciuria and hypercalcemia [6]. Phosphate supplements should be discontinued in patients with significantly increased parathyroid hormone levels [6].

Active vitamin D can be administered without phosphate supplements to adult patients with secondary hyperparathyroidism if close follow-up is performed [6].

It is suggested to supplement patients with native vitamin D (cholecalciferol or ergocalciferol) in case of vitamin D deficiency and to ensure normal calcium intake [6].

In patients undergoing medical therapy, monitoring and adjustment of treatment doses should be based on measurements of plasma and urine calcium and phosphate, creatinine, ALP, PTH and 25(OH) vitamin D at each visit [6, 7].

#### Treatment with burosumab

In 2018, the European Medicines Agency (EMA) granted conditional marketing authorization to the anti-FGF23 monoclonal antibody burosumab for the treatment of XLH in children aged ≥ 1 year with a growing skeleton and evidence radiography of bone diseases [19]. In late 2020, authorization was extended to adolescents and adults with XLH and radiographic evidence of bone disease, regardless of growth status. In March 2023, Italian Medicines Agency (AIFA) approved the indication of burosumab for adolescent and adult patients with XLH under the reimbursement regime by the National Health Service [20]. Based on the AIFA statement of March 13, 2023, the drug Crysvita^®^ (burosumab) is indicated for the treatment of XLH in patients over 12 years of age, with evidence of active disease (Rickets Severity Score ≥ 1.5 and until skeletal maturity is reached in subjects in whom epiphyseal welding has not already occurred; skeletal pain attributable to XLH and at least one active fracture/pseudofracture in adult subjects) and already subjected to conventional therapy with phosphate and/or analogues of vitamin D.

Already in 2019, international guidelines recommended considering treatment with burosumab in adults with XLH with the following characteristics: persistent bone and/or joint pain due to XLH and/or osteomalacia that limits daily activities; pseudo-fractures or fractures related to osteomalacia; insufficient or refractory response to conventional therapy [6]. Treatment with burosumab was also recommended if patients experience complications related to conventional therapy [6, 8].

The starting dose of burosumab is 1.0 mg/kg body weight (maximum dose of 90 mg), administered subcutaneously every 4 weeks [6, 21].

The dose should be discontinued if the fasting serum phosphate level increases above the upper limit of normal for age. Then, burosumab can be restarted at approximately half the previous dose when the serum phosphate concentration is below the normal range [6, 21].

It is recommended to avoid administering burosumab concomitantly with conventional therapy, when serum phosphate levels are within the normal range for age and in the presence of severe renal insufficiency [6].

#### Recommendations for musculoskeletal treatment

Interventions aimed at reducing bone and joint pain, deformity, stiffness, muscle weakness and improving walking distance and physical function are recommended. These interventions include the use of analgesics (for example, short periods of use of non-steroidal anti-inflammatory drugs), intra-articular infiltrations (in the presence of degenerative changes), physiotherapy, rehabilitation, physical activity, and non-pharmacological treatment of pain [6].

#### FGF23-independent hypophosphatemia

In addition to the FGF23-dependent forms of hypophosphatemia, there are other FGF23-independent forms, for example those linked to malabsorption or primary tubular hyporeabsorption of phosphate. The therapy to be used for these forms is different from that used for FGF23-dependent hypophosphatemia. For example, treatment for hereditary hypophosphatemic rickets with hypercalciuria (HHRH; OMIM: 241530) and hypophosphatemia secondary to Fanconi and Dent syndrome consists of long-term medical therapy with phosphate supplements, without vitamin D supplementation [22].

## Follow-up

### Definition of follow-up intervals

Patients with hypophosphatemia positively responding to treatment and/or in stable condition should be evaluated by a multidisciplinary team at least every 6 months [6, 8]. Adult patients with XLH should be seen every 6 months if receiving therapy, or once a year if not treated with drugs [6].

Dental visits are recommended twice a year after tooth eruption, to prevent and treat dental infections and periodontitis [6].

In patients receiving burosumab, it is recommended to monitor fasting serum phosphate levels along with maximal tubular phosphate reabsorption per glomerular filtration rate (TmP/GFR) every 2 weeks during the first month of treatment and every 4 weeks for the following 2 months and thereafter, as appropriate; it is also recommended to measure fasting serum phosphate level 4 weeks after dose adjustment and it is suggested to measure serum 1,25(OH)2 vitamin D levels every 6 months together with urinary calcium excretion [6, 8].

In patients receiving conventional treatment or burosumab, renal ultrasound is recommended at least every 2 years in patients without nephrocalcinosis and at annual intervals in patients with nephrocalcinosis and/or persistent hypercalciuria [6, 8].

A dental orthopantomography (X-ray of the upper and lower jaw and teeth) is recommended at 5 years of age and in adults with recent oral manifestations. Radiographs should be repeated based on individual needs; retro-coronal and periapical radiographs and cone beam computed tomography can be used to detect and monitor endodontic, periodontal, or peri-implant infections [6].

The follow-up interval should be individualized to the patient, with more regular follow-up in young or growing children (on average every 3 months) than in adults (who may be followed every 6 to 12 months, depending on depending on treatment, symptoms and needs) [6, 7].

In asymptomatic adult patients not receiving medical therapy, there is little need to repeat biochemical or radiological testing more than once a year. However, it is recommended to monitor 25(OH)D at least every twelve months, especially during winter, regardless of whether the patient receives medical treatment or not [7].

### Definition of blood tests

Monitoring of blood levels of alkaline phosphatase (ALP; total serum ALP levels in children and bone-specific ALP in adults), calcium, phosphate, creatinine, parathyroid hormone (PTH), and 25(OH) vitamin D is recommended [6, 8]. It is also recommended to monitor 1,25(OH)2D in patients receiving burosumab [6].

It is recommended to measure 24-hour urinary calcium excretion or urinary calcium and creatinine levels to estimate the urinary calcium/creatinine ratio in patients receiving conventional treatment or burosumab [6, 8].

### Definition of clinical tests

It is recommended to measure height, weight and blood pressure and calculate body mass index (BMI) [6].

It is also recommended to record the history of headache, oral manifestations (including periodontal disease, tooth abscesses or maxillofacial cellulitis), musculoskeletal pain, pseudo-fractures, fatigue and level of physical function [6].

It is also recommended to look for evidence of deafness, enthesopathies, arthrosis, spinal deformities and scoliosis, muscle deficit, range of motion, manifestations related to Chiari 1 malformation and/or intracranial hypertension [6].

Cranial magnetic resonance imaging (MRI) is recommended (if possible, including a “black bone” sequence for skull imaging) in case of skull morphology suggesting craniosynostosis or clinical signs of intracranial hypertension [6, 8].

Routine dual-energy X-ray absorptiometry (DXA) or peripheral quantitative computed tomography (pQCT) is not recommended in patients with XLH for assessment of bone health [6, 8].

It is suggested to perform the 6-minute walk test (6MWT) and evaluate the quality of life in patients aged 5 years and older at annual or biennial intervals [6, 8].

In the consensus of Laurent et al. [7] the following clinical tests are also recommended:

- height and growth velocity, signs or rickets (curvature of the limbs, chest, …), intermalleolar or intercondylar distance.
- dysmorphic features, head circumference and shape, craniosynostosis, signs of intracranial hypertension (fundoscopy if possible symptomatic).
- bone tenderness, joint mobility, spinal examination, enthesis.
- hearing evaluation.
- skin (nevi, café-au-lait spots).

### Delphi Study

**Fig. 1:**
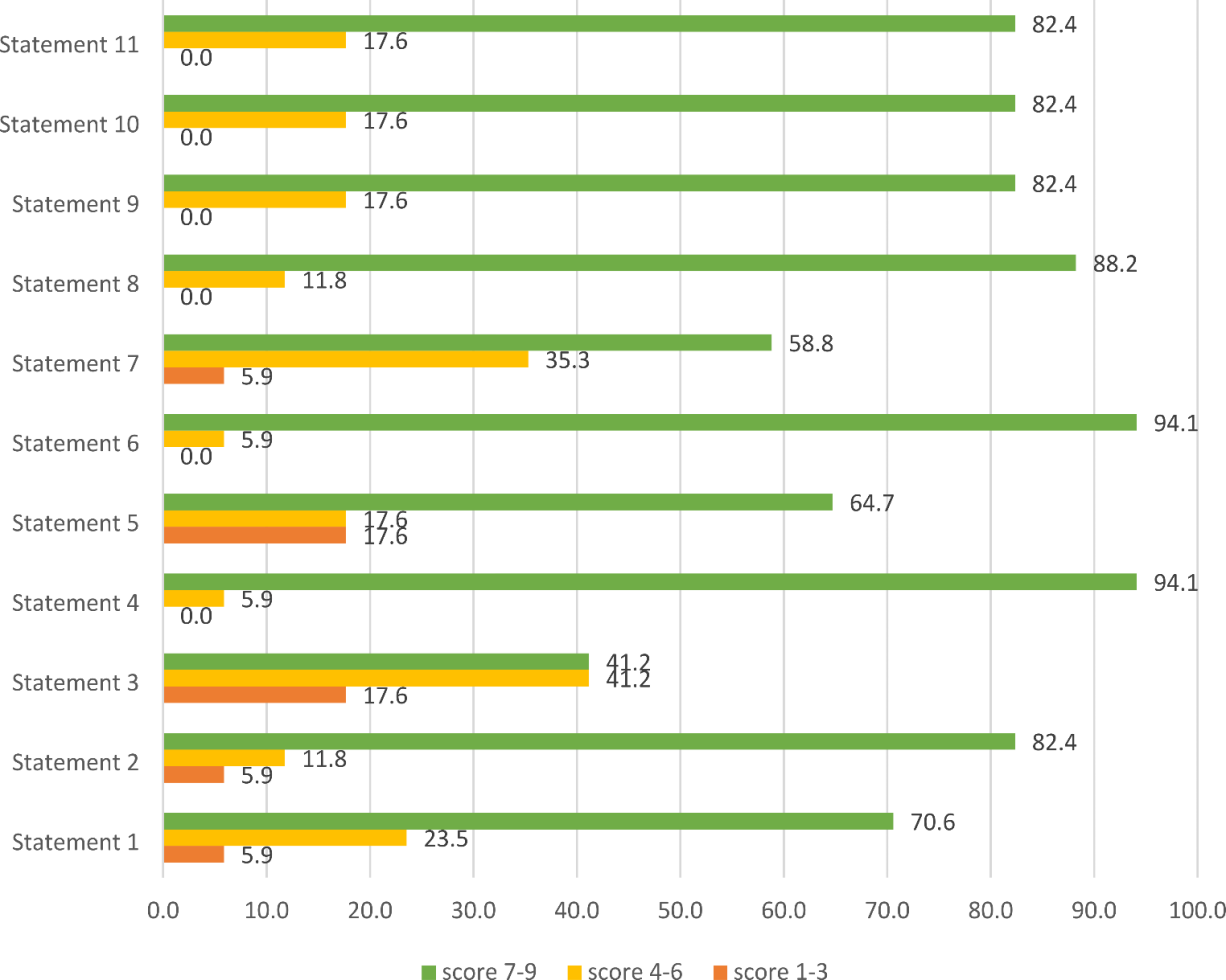
Scores of the proposed statements (first Delphi round). Results are presented in %

In the first round of the Delphi process, 17 out of the 25 clinical experts invited to participate responded (response rate 68%). Among the respondents, the majority were endocrinologists (*n* = 11); other respondents were internal medicine specialists (*n* = 4), one nephrologist and one rheumatologist. Strong convergence (over 70% consensus) was recorded for 8 of the 11 proposed statements (73%). Figure 1 shows the score distribution for all 11 statements. The 8 statements for which strong agreement was reached were S1, S2, S4, S6, S8, S9, S10, and S11. It is worthwhile to highlight that none of the statements had a score within the disagreement range.

Three statements, S3, S5, and S7, were submitted for the second round of the survey. In the first round, as for the S3 statement (“FGF23 measurement is considered necessary for the diagnosis and initiation of treatment of hypophosphatemia”), the same percentage of respondents (41.2%) expressed strong and moderate agreement, with a minority of respondents (17.6%) indicating no agreement. As for the S5 statement (“A vitamin D level < 30 ng/mL is an indication for its correction in patients with hypophosphatemia”), most of respondents (64.7%) expressed strong agreement, while the same percentage of respondents (17.6%) expressed moderate or no agreement. Finally, as for the S7 statement (“As part of a vegetarian/vegan diet, it is necessary to increase the intake of vegetables that provide a sufficient level of phosphate, such as bran, wheat germ, soy”), all but one respondent strongly or moderately agreed with the proposed statement.

**Fig. 2:**
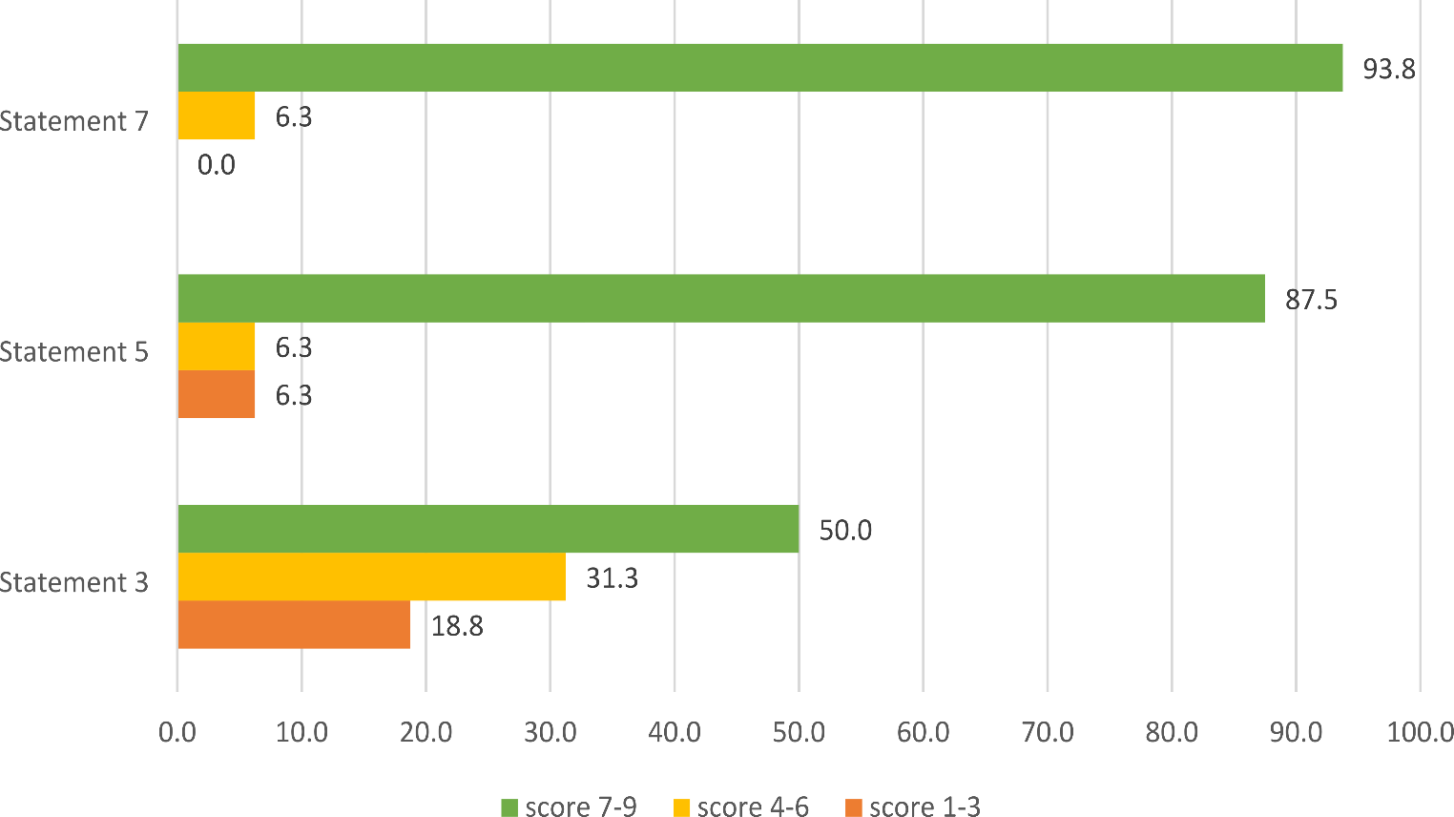
Scores of the proposed statements (second Delphi round). Results are presented in %

Among the 17 clinical experts who had answered in the first round, 16 completed the second-round survey (response rate: 94%). In the second round, responders were additionally requested to provide an explanation for any disagreement. Specifically, they were asked to justify any score that suggested disagreement with the proposed statement, (i.e., a score lower than 7), in order to assist the analysis of the responses. Strong agreement was recorded for two of the three re-proposed statements (Fig. 2). In conclusion, consensus was not reached only for one statement, namely the one related to the measurement of FGF23 for diagnostic purposes.

## Discussion

This Position Statement primarily relies on studies focused on XLH patients (5 out of 7 studies, as indicated in Table 2). However, it is recognized that in clinical practice many patients with hypophosphatemia may suffer from other genetic disorders or TIO. Therefore, we acknowledge that the recommendations provided in this Position Statement may not be universally applicable to all patients with hypophosphatemia. Nonetheless, given the lack of guidelines addressing other forms of hypophosphatemia, we believe that this Position Statement could still offer valuable insights for managing patients with other genetic disorders causing low phosphate levels or those with TIO.

The results of the consensus process were shared and discussed with the advisory board. It was observed that very high levels of agreement were reached on most statements (often above 80%). This indicates a general alignment of the panel participants with the statements proposed by the advisory board. Special attention was given to the statement for which it was not possible to reach consensus across the two rounds, i.e. the one labelled as S3, relating to the measurement of FGF23 for diagnostic purposes. This statement collected discordant opinions among the 16 respondents, with a slight consensus in favour: 50% of the respondents in fact expressed a high level of agreement (scores from 7 to 9) on the statement indicating the need to measure FGF23 for the diagnosis and initiation of hypophosphatemia treatment. Respectively, 31% and 19% of respondents reported to be “moderately in agreement” or “slightly or not at all in agreement” with this statement. From the analysis of the reasons provided by the respondents, it emerged that the unreached consensus might be due to a partial misunderstanding of the statement itself: in fact, the statement wording probably failed to clarify that the FGF23 measurement plays a crucial role exclusively in the evaluation of the hereditary forms of hypophosphatemia. The members of the advisory board agreed that clinical chemistry and laboratory tests can certainly, in the early stages of the diagnostic process, lead towards the exclusion of FGF23-independent forms of hypophosphatemia. It was therefore reasonably assumed that a reformulation of the statement that included a specification on the nature of the condition would have resulted in substantially different levels of agreement and potentially favourable opinions from the panel. An additional element that frequently emerged, extremely important from a perspective of optimization and provision of health services, is related to the availability of the FGF23 measurement test. In fact, FGF23 is carried out only in some highly specialized centres in our country- which however do not manage all patients. Finally, it was highlighted that, when available, the measurement of FGF23 is not reimbursed by the National Health Service in a relevant proportion of cases. However, in some regions, it is provided at no additional cost for the patient.

## Conclusions

Our results offer the opportunity to make relevant considerations on the management of hypophosphatemic patients. First, they highlight that the Italian community of clinical experts is well informed and generally in agreement concerning the management of hypophosphatemia in adult patients. Furthermore, the analysis strongly underlines the need to develop clear national guidance documents (e.g. consensus, guidelines), not only to support the clinicians (or the multidisciplinary team) in patient management, but also to provide informative elements to those who, on different levels, have the responsibility to define the pathways and related services for a more accurate management of adults with hypophosphatemia.

## Electronic supplementary material

Below is the link to the electronic supplementary material.

Supplementary Material 1

Supplementary Material 2

Supplementary Material 3

## Appendix 1: Panel of participating experts (*n* = 17)

**Table:**

Delphi panel member	Specialty	Affiliation
Maria Rosaria Ambrosio	Endocrinologist	Department of Medical Sciences, Section of Endocrinology, Geriatrics & Internal Medicine, University of Ferrara, Ferrara, Italy.
Elisa Cairoli	Endocrinologist	Istituto Auxologico Italiano IRCCS, Department of Endocrine and Metabolic Diseases, Milan, Italy.
Valentina Camozzi	Endocrinologist	UOC Endocrinologia, DIMED, Azienda Ospedaliera Università di Padova, Padova, Italy.
Salvatore Cannavò	Endocrinologist	Endocrine Unit, University Hospital G. Martino, University of Messina, Messina, Italy.
Cristina Eller-Vainicher	Endocrinologist	Unit of Endocrinology, Fondazione IRCCS Cà Granda-Ospedale Maggiore Policlinico, Milan, Italy.
Sandro Giannini	Internist	Clinica Medica 1, Department of Medicine, University of Padova, Padova, Italy.
Laura Gianotti	Endocrinologist	Division of Endocrinology and Diabetology, ASLCN1 Cuneo, Italy.
Andrea Giusti	Rheumatologist	Metabolic Bone Diseases Unit & Fracture Liaison Service, Department of Medical Specialties, Regional Health Trust 3, Genova, Italy.
Daniela Merlotti	Internist	Department of Medical Sciences, Azienda Ospedaliera Universitaria Senese, Siena, Italy.
Silvia Migliaccio	Endocrinologist	Department of Experimental Medicine, University Sapienza of Rome, Rome, Italy.
Salvatore Minisola	Internist	Prof. Onorario di Medicina Interna, “Sapienza” Università di Roma, Roma, Italy.
Vincenzo Montinaro	Nephrologist	UOC Nefrologia e Dialisi, Ente Ecclesiastico - Ospedale Generale Regionale “F. Miulli”, 70021 Acquaviva delle Fonti (BA), Italy.
Andrea Palermo	Endocrinologist	1) Fondazione Policlinico Universitario Campus Bio-Medico, Rome, Italy. 2) Unit of Metabolic Bone and Thyroid Disorders, Department of Medicine, Università Campus Bio-Medico di Roma, Rome, Italy.
Daniela Pasquali	Endocrinologist	Department of Advanced Medical and Surgical Sciences, University of Campania Luigi Vanvitelli, Naples, Italy.
Giovanni Passeri	Internist	Department of Medicine & Surgery-University of Parma, AOU Parma, 43126 Parma, Italy.
Massimo Procopio	Endocrinologist	Division of Endocrinology, Diabetology and Metabolic Diseases, Dept. of Medical Science, University of Turin, Turin, Italy.
Antonio Stefano Salcuni	Endocrinologist	Unit of Endocrinology and Metabolism, University-Hospital S. M. Misericordia, Udine, Italy.
