# Supplementary material for: Areas of uncertainty on the diagnosis, treatment, and follow-up of hypophosphatemia in adults: an Italian Delphi consensus
Source: J Endocrinol Invest. 2024 Oct 8;48(2):257–67. doi: 10.1007/s40618-024-02458-4 (PMC11785637; doi:10.1007/s40618-024-02458-4)
Supplement: Supplementary file 1 — Supplementary Material 1 [file 40618_2024_2458_MOESM1_ESM.pdf]

Online Resource 1. Literature search strategy.

PubMed, 13/07/2022		
Search	Query	Results
#1	hypophosphatemia OR hypophosphatemic OR hypophosphataemia OR hypophosphataemic	7,643
#2	guideline[Title] OR guidelines[Title] OR consensus[Title] OR systematic review[Title] OR meta-analysis[Title]	373,619
#3	#1 AND #2	63
#4	hypophosphatemia OR hypophosphatemic<br>Filters: Consensus Development Conference, Consensus Development Conference, NIH, Guideline, Meta-Analysis, Systematic Review	63
#5	#3 OR #4	81

Embase, 13/07/2022		
Search	Query	Results
#1	'hypophosphatemia'/exp OR 'hypophosphatemia' OR hypophosphataemia OR hypophosphatemic OR hypophosphataemic	16,817
#2	guideline:ti OR guidelines:ti OR consensus:ti OR 'systematic review':ti OR 'meta-analysis':ti	458,800
#3	#1 AND #2	177

Web of Science, 13/07/2022		
Search	Query	Results
#1	TS=(hypophosphatemia OR hypophosphataemia OR hypophosphatemic OR hypophosphataemic)	7,052
#2	TI=(guideline OR guidelines OR consensus OR "systematic review" OR "meta-analysis")	454,524
#3	#1 AND #2	77

Google Scholar, 13/07/2022		
Search for international guidelines, consensus statements, and systematic reviews		
Search	Query	Results
#1	(hypophosphatemia OR hypophosphatemic OR hypophosphataemia OR hypophosphataemic) AND (guideline OR guidelines OR consensus OR systematic review OR meta-analysis)	The first 100 records were examined.

Google Scholar and Google, 13/07/2022		
Search for Italian guidelines and consensus statements		
Search	Query	Results
#1	(ipofosfatemia OR ipofosfatemico OR ipofosfatemica OR ipofosfatemici OR ipofosfatemiche OR ipofosforemia OR ipofosforemie OR ipofosforemico OR ipofosforemica OR ipofosforemici OR ipofosforemiche) AND (linea guida OR linee guida OR consensus)	The first 30 Google Scholar records and the first 30 Google records were examined.

Google Scholar and Google, 13/07/2022		
Search for Italian integrated care pathways		
Search	Query	Results
#1	(ipofosfatemia OR ipofosfatemico OR ipofosfatemica OR ipofosfatemici OR ipofosfatemiche OR ipofosforemia OR ipofosforemie OR ipofosforemico OR ipofosforemica OR ipofosforemici OR ipofosforemiche) AND (PDTA OR percorso diagnostico terapeutico assistenziale)	The first 30 Google Scholar records and the first 30 Google records were examined.
