# Supplementary material for: Areas of uncertainty on the diagnosis, treatment, and follow-up of hypophosphatemia in adults: an Italian Delphi consensus
Source: J Endocrinol Invest. 2024 Oct 8;48(2):257–67. doi: 10.1007/s40618-024-02458-4 (PMC11785637; doi:10.1007/s40618-024-02458-4)
Supplement: Supplementary file 2 — Supplementary Material 2 [file 40618_2024_2458_MOESM2_ESM.pdf]

**PRISMA 2020 flow diagram for new systematic reviews which included searches of databases and registers only**

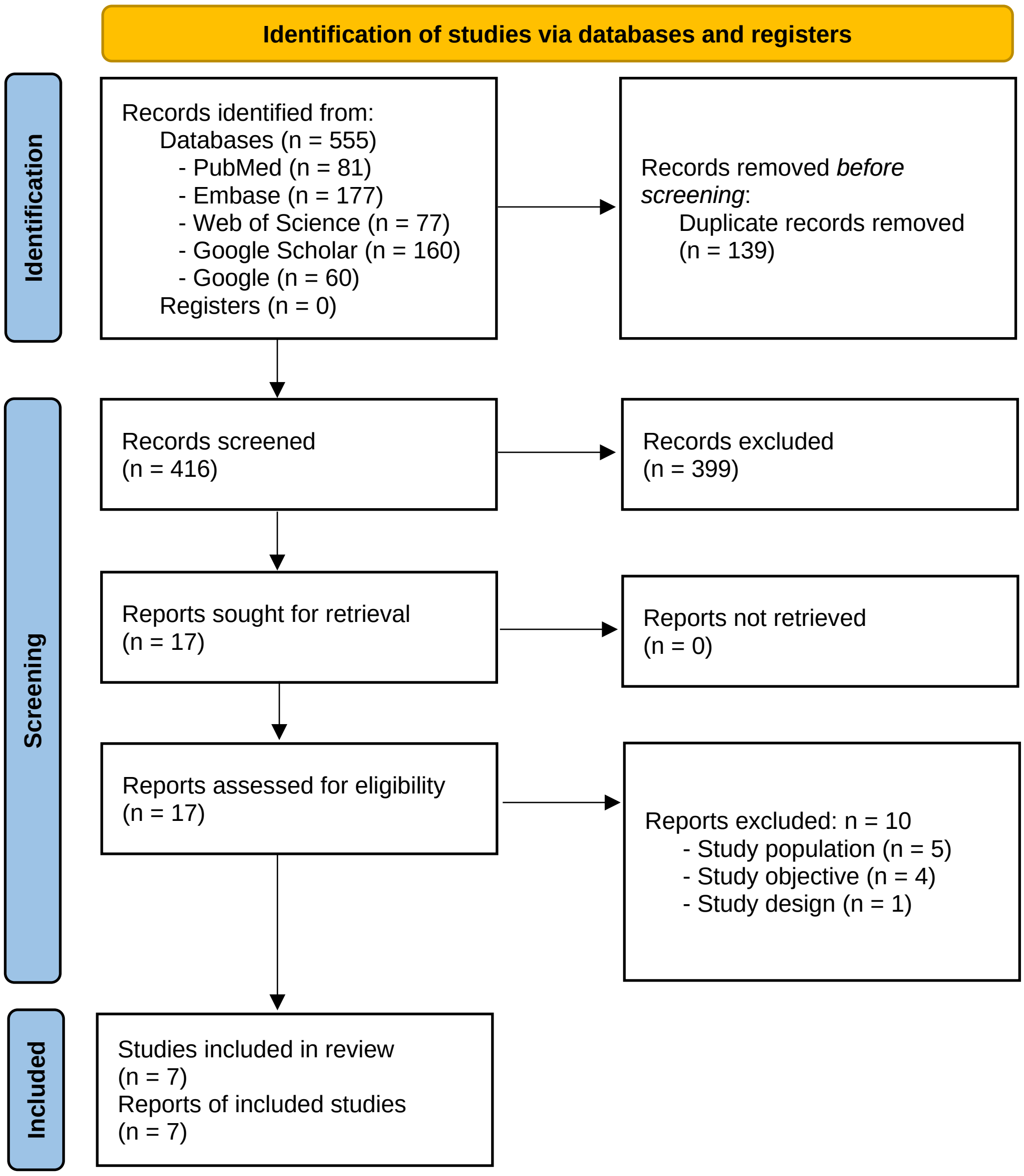


*From:* Page MJ, McKenzie JE, Bossuyt PM, Boutron I, Hoffmann TC, Mulrow CD, et al. The PRISMA 2020 statement: an updated guideline for reporting systematic reviews. BMJ 2021;372:n71. doi: 10.1136/bmj.n71

For more information, visit: http://www.prisma-statement.org/
