# Supplementary material for: Areas of uncertainty on the diagnosis, treatment, and follow-up of hypophosphatemia in adults: an Italian Delphi consensus
Source: J Endocrinol Invest. 2024 Oct 8;48(2):257–67. doi: 10.1007/s40618-024-02458-4 (PMC11785637; doi:10.1007/s40618-024-02458-4)
Supplement: Supplementary file 3 — Supplementary Material 3 [file 40618_2024_2458_MOESM3_ESM.pdf]

Online Resource 3. List of excluded studies.

References	Reason for exclusion
Al Juraibah F, Al Amiri E, Al Dubayee M, Al Jubeh J, Al Kandari H, Al Sagheir A, Al Shaikh A, Beshyah SA, Deeb A, Habeb A, Mustafa M, Zidan H, Mughal MZ. Diagnosis and management of X-linked hypophosphatemia in children and adolescent in the Gulf Cooperation Council countries. Arch Osteoporos. 2021 Mar 4;16(1):52. doi: 10.1007/s11657-021-00879-9.	Population: children and adolescents with XLH.
Padidela R, Cheung MS, Saraff V, Dharmaraj P. Clinical guidelines for burosumab in the treatment of XLH in children and adolescents: British paediatric and adolescent bone group recommendations. Endocr Connect. 2020 Oct;9(10):1051-1056. doi: 10.1530/EC-20-0291.	Population: children and adolescents with XLH.
Sandy JL, Simm PJ, Biggin A, Rodda CP, Wall CL, Siafarikas A, Munns CF. Clinical practice guidelines for paediatric X-linked hypophosphataemia in the era of burosumab. J Paediatr Child Health. 2022 May;58(5):762-768. doi: 10.1111/jpc.15976.	Population: children and adolescents with XLH.
Smith S, Remmington T. Recombinant growth hormone therapy for X-linked hypophosphatemia in children. Cochrane Database Syst Rev. 2021 Oct 7;10(10):CD004447. doi: 10.1002/14651858.CD004447.pub3.	Population: children and adolescents with XLH.
Reintam Blaser A, Gunst J, Ichai C, Casaer MP, Benstoem C, Besch G, Dauger S, Fruhwald SM, Hiesmayr M, Joannes-Boyau O, Malbrain MLNG, Perez MH, Schaller SJ, de Man A, Starkopf J, Tamme K, Wernerman J, Berger MM. Hypophosphatemia in critically ill adults and children - A systematic review. Clin Nutr. 2021 Apr;40(4):1744-1754. doi: 10.1016/j.clnu.2020.09.045.	Population: critically ill adults and children with hypophosphatemia admitted to intensive care units.
González-Lamuño D, Lorente Rodríguez A, Luis Yanes MI, Marín-Del Barrio S, Martínez Díaz-Guerra G, Peris P. Clinical practice recommendations for the diagnosis and treatment of X-linked hypophosphatemia: A consensus based on the ADAPTE method. Med Clin (Barc). 2022 Aug 12;159(3):152.e1-152.e12. English, Spanish. doi: 10.1016/j.medcli.2021.07.029.	Study objective: adaptation of a clinical guideline (Haffner D, Emma F, Eastwood DM, Duplan MB, Bacchetta J, Schnabel D,et al. Clinical practice recommendations for the diagnosis and manage-ment of X-linked hypophosphataemia. Nat Rev Nephrol. 2019;15:435–55).
McDermott M, Twomey P, Van der Kamp S, Crowley RK. X-linked hypophosphatemia patient work-up at St. Vincent's University Hospital is managed to European consensus guidelines. Irish Endocrine Society 44th Annual Meeting. Ir J Med Sci. 2021 Apr;190(Suppl 3):S94-S95. doi: 10.1007/s11845-021-02557-8.	Study objective: audit to assess the compliance at St. Vincent's University Hospital with clinical practice guidelines (Haffner et al, 2019) when assessing patients with XLH.
Padelli M, Leven C, Sakka M, Plée-Gautier E, Carré JL. Causes, conséquences et traitement de l'hypophosphorémie : une revue systématique de la littérature [Causes, consequences and treatment of hypophosphatemia: A systematic review]. Presse Med. 2017 Nov;46(11):987-999. French. doi: 10.1016/j.lpm.2017.09.002.	Study design: narrative review.
Seefried L, Smyth M, Keen R, Harvengt P. Burden of disease associated with X-linked hypophosphataemia in adults: a systematic literature review. Osteoporos Int. 2021 Jan;32(1):7-22. doi: 10.1007/s00198-020-05548-0.	Study objective: to evaluate the burden of XLH in adults.
Turan S. Guidelines on the diagnosis and management of X-linked hypophosphatemia. J Ist Faculty Med 2020;83(Suppl.1):S1-S16. doi: 10.26650/IUITFD.2020.0200	Study objective: adaptation of a clinical guideline (Haffner et al, 2019); article in Turkish.
